# The effects of exergaming on executive functions in children with ADHD: a systematic review and meta-analysis of randomised controlled trials

**DOI:** 10.1080/21642850.2026.2614157

**Published:** 2026-01-16

**Authors:** Febryansah Gilang Aris Pradana, Rakhmat Ari Wibowo, Graham Baker

**Affiliations:** a Faculty of Sports Science and Health, Universitas Negeri Surabaya, Surabaya, Indonesia; b Physical Activity for Health Department, The University of Edinburgh, Edinburgh, Scotland, UK

**Keywords:** Serious games, cognitive function, physical activity, disability, neurodevelopmental disorders

## Abstract

**Purpose:**

A growing number of studies have investigated the effectiveness of exergaming on the executive functions (EFs) in children with ADHD. Whilst some studies have shown beneficial effects, other studies provide mixed effects and there has yet to be a focused synthesis of the literature on this topic.

**Methods:**

A systematic review (PROSPERO; CRD42024549395) was conducted following PRISMA guidance, drawing from eligible studies across eleven databases. Studies were screened based on eligibility criteria by two independent reviewers. Risk of bias (Cochrane ROB2) and certainty of evidence (GRADE criteria) were independently assessed by two reviewers. The Synthesis Without Meta-analysis (SWiM) using vote counting based on direction of effect was conducted on studies with incomplete data. Separate meta-analyses were performed for each EF process using random-effect meta-analysis.

**Results:**

A total of 2,263 studies were identified. After removing duplicates and non-RCTs automatically, 1,546 studies were screened by title and abstract resulting in 35 full texts being retained for detailed screening with 4 studies meeting all inclusion criteria. Two studies in the SWiM showed beneficial effects on overall EFs. Meta-analyses on two studies found exergaming was beneficial for inhibition (SMD = −5.07; 95%-CI [−14.08, 3.95]), working memory (SMD = −0.31; 95%-CI [−0.88, 0.26]), and cognitive flexibility (SMD = −0.84; 95%-CI [−1.50, −0.19]). The mean drop-out rate was 13.82% indicating high adherence. Implementation data identified that a console, game license, a room, television, and supervision from trainers/parents are needed to implement exergaming across different settings and contexts.

**Conclusion:**

The results indicate that although the direction of effects shows positive trends, the evidence remains insufficient and imprecise to determine the clear conclusion whether exergaming can increase EFs and adherence to PA in children with ADHD. Future research with high-quality measurement of the outcome and completely reporting outcome data is necessary to strengthen the evidence and to inform the implementation.

## Introduction

One of the most common neurodevelopmental disorders that affects children is attention-deficit/hyperactivity disorder (ADHD) with an estimated global prevalence of 7.6% (Salari et al., 2023). ADHD has three main symptoms which are inattention, hyperactivity/impulsivity, or a combined presentation (American Psychiatric Association, 2022). These symptoms have been closely linked with a reduction in executive functions (EFs) (Nigg et al., 2002; Willcutt et al., 2005) and usually appear in children (Colomer et al., 2017). EFs regulate fundamental cognitive operations; therefore, EFs are indispensable for behavioural flexibility, adaptation, and goal orientation (Diamond, 2013). EFs comprise three key processes—inhibition, working memory, and cognitive flexibility—profoundly involved in children’s learning and academic achievement (Biederman et al., 2004). Hence, due to the reduction in EFs in children with ADHD, they commonly struggle with inadequate performance and academic achievement (Arnold et al., 2020).

Medication has become the most frequent treatment to address executive dysfunctions in children with ADHD (Barbaresi et al., 2013). However, the use of medication potentially introduces side effects such as headaches, anxiety, and sleep problems (Briars & Todd, 2016; Lim et al., 2008). Cognitive training is an alternative approach to treat executive dysfunctions; however, the repetitive training can leave children with ADHD feeling dull and exhausted when performing cognitive training particularly if the training lacks reinforcement (Luman et al., 2005). Those motivational problems could reduce the effectiveness of cognitive training (Prins et al., 2011).

An alternative approach to addressing executive dysfunction is through engagement in physical activity (PA) (Chan et al., 2022; Welsch et al., 2021). The World Health Organisation (WHO, 2018) suggests that children with ADHD will obtain favourable results on EFs if they engage in moderate-to-vigorous PA at least 60 minutes a day or 3 days a week of vigorous PA. However, although the benefits of engaging in PA for children with ADHD are known, adherence to PA in children with ADHD is typically lower compared with those without ADHD (Mercurio et al., 2021). Low motivation and forgetfulness become barriers to adherence in PA for children with ADHD because PA is often perceived as fatiguing and uninteresting (Boman & Bernhardsson, 2023; Ogrodnik et al., 2023).

Therefore, providing an additional adjunct or independent treatment for children with ADHD may aid in the effectiveness of interventions which aim to increase levels of PA. Halperin and Healey (2011) contended that the combination of PA and cognitive challenges can be an effective treatment to improve executive dysfunctions and low adherence to PA in children with ADHD. Concurrently, Moreau and Conway (2014) assert that the most significant impacts on EFs in children with ADHD can be achieved by amalgamating PA and cognitive training. One of the options to implement a cognitive element combined with an active approach is through exergaming.

Exergaming is an innovative concept that incorporates exercise with gaming. Exergaming requires players to move their bodies in the same ways as the ones in the digital games; hence, the players will acquire the benefits of engaging in PA (Wijffelaars & Markopoulos, 2023) whilst being cognitively challenged. For instance, a player who plays Wii Sports baseball games will hold a Wii Remote with motion-sensing and make arm swing movements at the same time as ball movement in the game; hence, the results in the game will be the baseball player swings the baseball bat and hits the ball (Miyachi et al., 2010). It has been proposed that exergaming can be an enjoyable exercise because of the game-field strategy, can accelerate motivation and EFs, and can combat low adherence to PA in children without ADHD (Primack et al., 2012; Staiano & Calvert, 2011).

Therefore, developing a robust evidence base of the effectiveness of exergaming to increase EFs and adherence to PA in children with ADHD is essential. A growing number of studies have investigated the effectiveness of exergaming on the EFs in children with ADHD and found positive results (Benzing & Schmidt, 2019; Chang et al., 2022). However, the effects on the outcomes of those studies are mixed. For example, Benzing and Schmidt (2019) found that exergaming has moderate effects on inhibition (*d* = 0.58) and cognitive flexibility (*d =* 0.65), but no effect on working memory (*d* = 0.02). In contrast, Chang et al. (2022) found that exergaming has large effects on overall EFs (*d* = 0.96).

However, to date, there has yet to be a focussed synthesis of the literature on this topic. One systematic review and meta-analysis, related to this topic but broader, examined the effects of technology-based interventions. Conducted by Wong et al. (2023), this review found promising effects on increasing EFs in children with ADHD; however, only one of the 19 included studies specifically used exergaming as the main intervention approach. Moreover, the results introduced substantial heterogeneity (I^2^ = 52.7%) even after considering moderators, including sample size, type of control group, number of sessions, game element, and settings. The complementary effect of exercise components within the exergame might be the source of heterogeneity which still need to be elucidated (Glasziou & Sanders, 2002; Song et al., 2023). A more recent review provided pooled effect sizes of exergaming on EFs among children with ADHD but incorporated both acute and chronic effects of exergaming which limit its applicability and generalisability (Kou et al., 2024). Therefore, the effects of exergaming specifically to improve EFs in children with ADHD remain unclear and with an emerging evidence base, a synthesis is warranted. Furthermore, since adherence to PA in children with ADHD is typically low, examining the adherence to exergaming interventions is essential to provide accurate recommendations of the utility of exergaming to increase PA in children with ADHD (Gao & Chen, 2014). Moreover, providing this evidence on how exergaming interventions could be implemented by current service providers, either school or health-care providers, and parents, as parts of the comprehensive management of children with ADHD would help in translating the evidence into real-world practice (Chalkidou & Anderson, 2009; Estabrooks & Glasgow, 2006; Evans et al., 2017; Wolraich and Hagan, 2019).

Thus, the aim of this review is to synthesise the current literature base on the effects of exergaming to increase EFs in children with ADHD, and to examine adherence to, and implementation of the interventions which have been delivered in this area. The specific research questions were as follows:

1. What is the effectiveness of exergaming on executive functions in children with ADHD?
2. What is the adherence of children with ADHD to exergaming interventions?
3. What is the implementation strategy for exergaming interventions to increase executive functions in children with ADHD?

## Methods

This systematic review followed the Preferred Reporting Items for Systematic Review and Meta-Analyses (PRISMA) checklist (Page et al., 2021; supplementary material *S1*) and was guided by Cochrane Guidelines for Systematic Reviews (Higgins et al., 2023). The protocol was registered in the International Prospective Registers of Systematic Reviews (PROSPERO; CRD42024549395; Pradana et al., 2024). This systematic review was not subject to ethical approval.

### Eligibility criteria

Studies were selected based on specified inclusion and exclusion criteria using the Population, Intervention, Control, Outcomes, and Study design (PICOS) framework as presented in Table 1. Only English-language published articles were included. There were no date or location restriction criteria to ensure the search was as inclusive as possible (Lefebvre et al., 2025).

**Table 1.** Eligibility criteria.

Categories	Inclusion and Exclusion
Population	Children with ADHD aged 4 **–** 12 years old (the age range for children provided by the National Health Service (NHS, 2021) who have been diagnosed using the standardised diagnostic tool, for example the Diagnostic and Statistical Manual of Mental Disorders (DSM) or the International Classification of Diseases (ICD) criteria.
Intervention	Inclusion: Studies that used exergaming as at least one of their interventions, for example exergaming + cognitive training interventions. Indoors or outdoors, any structured (planned exergaming or supervised by a trainer) or semi-structured (free play) exergaming interventions. All domains of exergaming: rehabilitation, preventive healthcare, and active entertainment/fitness games. Exclusion: Single-bout, acute intervention studies in order to investigate chronic adaptation (longer effects) since chronic exercise intervention has significant moderate-to-large effects on EFs compared with acute exercise (Liang et al., 2021).
Control	Inclusion: 1. Exergaming intervention vs control (e.g. no treatment, waiting list control). 2. Exergaming intervention vs non-active alternative treatment (e.g. usual care, cognitive training). 3. Exergaming intervention + non-active treatment vs non-active treatment. Exclusion: Studies that used PA as their control group, or if the control condition is not appropriate (comparative studies where all arms receive a variation of an exergaming intervention), or do not have a control condition (e.g. single-group studies, cross-over trials).
Outcomes	Overall EFs or each domain of EFs (inhibition, working memory, and cognitive flexibility) and adherence to exergaming interventions.
Study Type	Randomised Controlled Trials (RCTs) as the most robust and rigorous method to investigate the effects of intervention (Bhide et al., 2018).

### Information sources

A systematic search was conducted on May 19^th^—24^th^ 2024 on 11 electronic databases: MEDLINE (Ovid); CINAHL (EBSCO); CENTRAL; Embase (Ovid); PsycINFO (Ovid); SPORTDiscus (EBSCO); Scopus (Elsevier); Web of Science (Clarivate); ACM Digital Library; IEEE Xplore Digital Library; and Sport Medicine & Education Index.

### Search strategy

The search strategy was developed based on five categories from the PICOS (Table 2). The alternative words and truncation symbols were used to increase the optimal number of search terms and to catch the five search categories. Then, the five search categories were combined using the Boolean operator ‘AND’. The search strategy for the MEDLINE database is provided in Table 2 and was adapted for other databases. Backward and forward citation tracking of all included studies was conducted (Hirt et al., 2024) using the Citation Chaser tool (https://estech.shinyapps.io/citationchaser/) to improve search comprehensiveness and efficiency in time and resources (Haddaway et al., 2021; Haddaway et al., 2022).

**Table 2.** List of search strategies.

Categories	Terms
Children	child* OR kid* OR youth OR paediatric
ADHD	ADHD OR ‘attention deficit hyperactivity disorder’ OR ‘attention deficit disorder’ OR ‘attention deficit-hyperactivity disorder’ OR ‘attention-deficit/hyperactivity disorder’ OR ‘hyperactivity disorder’ OR ‘hyperkinetic syndrome’ OR ‘minimal brain damage’ OR ‘minimal brain dysfunction’ OR ‘neurodevelopmental disorder’
Exergame	exergam* OR video gam* OR game* OR gaming OR ‘active video games’ OR ‘computer games’ OR ‘online gam*’ OR ‘internet gam*’ OR ‘virtual reality’ OR vr OR ‘augmented reality’ OR ‘immersive virtual reality’ OR ‘immersive vr’ OR ivr OR wii OR ‘nintendo wii’ OR ‘wii gam*’ OR ‘wii fit’ OR ‘virtual cycl*’ OR ‘xbox kinect’ OR ‘physical action gam*’ OR ‘serious game’
Executive functions	‘executive function*’ OR ‘executive dysfunction’ OR ‘cognitive function*’ OR ‘executive abilit*’ OR ‘cognitive ability*’ OR ‘cognitive control’ OR ‘executive process*’ OR cognit* OR memor* OR vigilance OR distractibility OR ‘cognitive flexibility’ OR impulsiv* OR inhibit* OR ‘dual task’ OR ‘inhibitory control’ OR ‘working memory’ OR ‘short term memory’ OR ‘interference control’ OR ‘speed reaction’ OR ‘processing speed’ OR ‘reaction time’ OR ‘cognitive dysfunction’ OR ‘cognition disorders’
RCTs	‘randomised controlled trial’ OR rct OR ‘randomised controlled trial’

### Selection process

Covidence systematic review software (2024) (http://www.covidence.org/) was used to manage the screening process. After excluding duplicates, two reviewers (FGAP and RAW) independently screened the titles and abstracts of all retrieved studies based on the eligibility criteria to eliminate selection bias (McDonagh et al., 2013). The reviewers then independently screened the full text of selected studies to identify the final eligible studies to be included in the synthesis. Disagreements during title-abstract and full-text screening were resolved through discussion. The arbiter (third author; GB) mediated the discussion for discrepancies that could not be resolved. The PRISMA diagram was used to manage the detailed records of the stages (Page et al., 2021).

### Data collection process

Two researchers (FGAP and RAW) independently extracted the data to increase accuracy and minimise error (Buscemi et al., 2006). The extracted data, stored in Excel, included: study characteristics (author, year, title, country, study setting); participant characteristics (age, number, gender, type of ADHD, ADHD severity, diagnostic tool); intervention details (type of exergame, exergame features, duration, frequency and length of sessions, delivery mode, components of fitness); control condition; confounding variables; outcomes (measurement tool, unit of measurement, EFs domains, adherence, statistical results); and implementation strategy (brief description of intervention, context, economic, barriers, facilitators, description of implementation strategy, resource use, cost use, fidelity, important harms or unintended effects in each group) based on the combination of the Consensus on Exercise Reporting Template (CERT) (Slade et al., 2016), the Consolidated Standards of Reporting Trial-EHEALTH checklist (Eysenbach, 2011), and the Standards for Reporting Implementation Studies (StaRI) checklist (Pinnock et al., 2017). PlotDigitizer (2024) (https://plotdigitizer.com/), an online software to extract numerical data from images, were used to extract IQR data because the tool has high validity and reliability (Aydin & Yassikaya, 2022). Mean of extracted results from the two researchers were used. Any discrepancies were discussed and mediated by the arbiter (GB) when disagreements could not be resolved. The first author (FGAP) contacted the study authors via e-mail for studies with missing or unclear data.

### Data items

The primary outcome of this study was EFs (overall EFs, inhibition, working memory, and cognitive flexibility). The reviewers included studies that investigated changes in EFs between baseline and the end of intervention which used standardised outcome measures assessing either EFs or any specific process of EFs as a continuous outcome. For example, Benzing and Schmidt (2019) used computer-based tests using E-Prime Software, while Chang et al. (2022) used The Wisconsin Card Sorting Test. To prevent multiplicity, when a study used multiple instruments to measure EF outcomes, only one outcome was selected per study based on the following decision rule hierarchy: the stated primary outcome; the outcome used in any stated sample size calculation; the outcome reported in the abstract; the first outcome reported in the Results section (Baker et al., 2023).

The secondary outcome of this study was the adherence to the exergaming interventions measured through the drop-out rate in the exergaming intervention group. Moreover, the implementation strategy of exergaming was another outcome. The reviewers elicited the data from studies that investigated the implementation strategy or summarised intervention studies which could imply how exergaming could be implemented across different contexts and settings.

### Study risk of bias assessment

Two reviewers (FGAP and RAW) independently assessed the risk of bias (RoB) to increase accuracy and reliability of the process (Higgins et al., 2019). Clinical trial registries were tracked. Moreover, the Cochrane Risk of Bias tool for RCTs—RoB 2 was used because the tool is comprehensive and has clear guidance on how to assess and rate the risk of bias (De Cassai et al., 2023; Sterne et al., 2019). The researchers assessed bias: arising from the randomisation process; due to deviations from intended interventions (using intention-to-treat analysis); due to missing outcome data; in the measurement of the outcome; and in the selection of reported results (Sterne et al., 2019). The reviewers did not assess whether the participants and personnel were blinded to the group allocation in the RCT studies since this would not be suitable for the exergaming intervention study.

Discrepancies were discussed, and the arbiter (GB) mediated the discussion when disagreements could not be resolved. We presented a ‘Risk of bias’ table for all included studies using the *robvis* tool (McGuinness & Higgins, 2021). The reviewers assessed each domain in the RoB 2 using a rate of ‘low’ or ‘some concerns’ or ‘high’ risk of bias.

### Effect measures

The standardised mean difference (SMD) was calculated using the mean difference (MD) and the standard deviation (SD) resulting from the change from baseline for each outcome measure if there are at least two eligible studies to pool in the meta-analysis. When the studies did not report using change from baseline, the reviewers obtained the SD of the change from baseline by imputing it from available data (IQR) (Deeks et al., 2019). The reviewers used a tool (https://www.math.hkbu.edu.hk/~tongt/papers/median2mean.html) by Shi et al. (2023) to check the skewness of the IQR data, to estimate the mean of the sample from IQR data (Luo et al., 2018), and to estimate SD of the sample from IQR data (Wan et al., 2014).

### Synthesis methods

The authors further specified the narrative synthesis that was stated in the protocol (Pradana et al., 2024) by using The Synthesis Without Meta-analysis (SWiM) guideline using vote counting based on direction of effect (Campbell et al., 2020; supplementary material *S2*) for studies that did not report complete data to undertake a meta-analysis of effect estimates. The studies were grouped based on the outcomes: overall EFs; inhibition; working memory; and cognitive flexibility. The results of vote counting used three categorisations (positive direction of effect; negative direction of effect; and no clear effect).

Separate meta-analyses on at least two eligible studies to pool were performed for the different processes of EFs (inhibition, working memory, and cognitive flexibility) by pooling the appropriate data from included studies using Review Manager 5.4.1 (RevMan 5.4.1; Review Manager, 2020). Because the variance of the intervention design and unit of measurement is high, random-effects meta-analyses were conducted (Cumming, 2013).

SMD, *p*-values, and 95% confidence intervals (CI) were reported for all overall outcomes. Effect sizes were interpreted following guidelines from Cohen, (1988), with negative effect sizes indicating beneficial effects. The significance level was set to *α* < 0.05.

The heterogeneity was assessed by calculating the I^2^ statistics (Higgins & Thompson, 2002), with interpretation following the Cochrane guidelines (Higgins et al., 2011) and was presented as part of the forest plots in Review Manager. Because limited studies were included in the meta-analyses, subgroup, sensitivity analysis, meta-regression could not be performed (Higgins et al., 2011).

### Reporting bias assessment

Publication bias could not be assessed using a funnel plot due to the number of included studies being less than 10 (Page, 2018); therefore, we examined this by exploring registered trials and asking the study authors about the progress of their studies.

### Certainty assessment

Two researchers (FGAP and RAW) independently assessed the certainty of evidence for each outcome (overall EFs, inhibition, working memory, and cognitive flexibility) (Churchill et al., 2022), using the Grading of Recommendation, Assessment, Development and Evaluation (GRADE) via GRADEpro GDT (2024) (https://www.gradepro.org/). The reviewers assessed GRADE based on the risk of bias, inconsistency, indirectness, imprecision, publication bias, and large effect and then created informative statements based on the overall quality of evidence (Schünemann et al., 2023; Santesso et al., 2020). Discrepancies were discussed, and the arbiter (GB) mediated the discussion when disagreements could not be resolved.

## Results

### Study selection

A total of 2,263 studies were identified from electronic databases. After removing duplicates and non-RCTs automatically, 1,546 studies were screened for title and abstract resulting in 35 full texts being retained for detailed screening with 4 studies meeting all inclusion criteria. Following that, forward and backward citation searches were conducted resulting in an additional 308 studies identified; however, no additional study met inclusion criteria. Therefore, co-citing and co-cited citation searches could not be performed. There was no additional paper by the same author since all published exergaming studies by the same author were identified by databases.

Studies were excluded after full-text screening: four studies did not use eligible participants, seven studies did not use RCTs, two studies did not use eligible control groups, and 23 studies did not use exergaming interventions. One study (Valtr et al., 2023) almost met all inclusion criteria, but after detailed evaluation, the study was excluded due to ineligible intervention in which only a skilled model performed exergaming of the dart-throwing, while the participants watched the split-screen video of the trained model.

**Figure 1.**
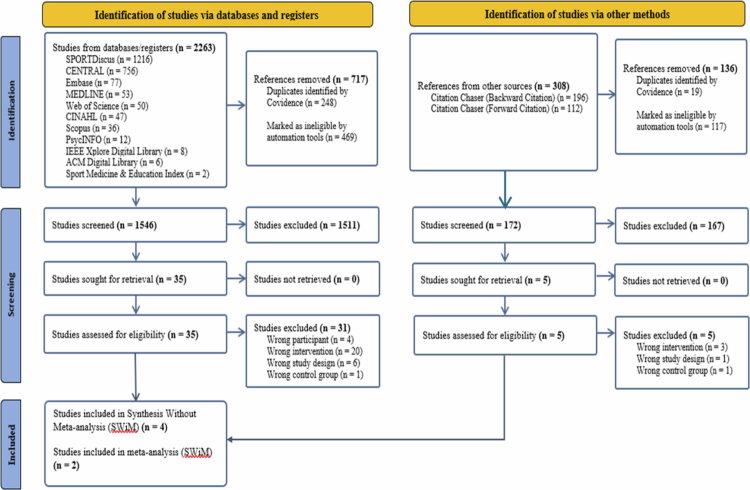
PRISMA flow diagram.

Out of 4 eligible studies, only one study (Zhao et al., 2024) reported complete outcome data for meta-analysis. After contacting three authors via email, data were obtained for an additional study (Benzing & Schmidt, 2019). Figure 1 shows a complete outline of the PRISMA flow diagram.

### Study characteristics

Table 3 summarises the study characteristics (full details available in supplementary material *S3*). The studies were conducted in three countries including Switzerland (*n* = 1) (Benzing & Schmidt, 2019), Taiwan (*n* = 1) (Chang et al., 2022), and China (*n* = 2) (Chu et al., 2023; Zhao et al., 2024) across four different settings; home (Benzing & Schmidt, 2019), school (Chang et al., 2022), laboratory (Chu et al., 2023), and hospital (Zhao et al., 2024). There was a total of 241 participants (192 boys), with sample sizes at baseline ranging from 32 (Chang et al., 2022) to 80 (Zhao et al., 2024). The mean age of the participants ranged from 5 to 10 years old.

**Table 3.** Study characteristics.

		Intervention		Control		
Study	Setting	*n*	*Mean age (SD)*	*n*	*Mean age (SD)*	Diagnostic tool
Benzing and Schmidt (2019)	Home	28	10.46 (1.30)	23	10.39 (1.44)	ICD-10
Chang et al. (2022)	School	16	8.38 (1.20)	16	8.38 (1.31)	DSM-5
Chu et al. (2023)	Laboratory	39	5.00 (0.82)	39	5.03 (0.83)	DSM-5
Zhao et al. (2024)	Hospital	40	8.5 (1.5)	40	8.3 (1.1)	DSM-5

### Intervention characteristics

Table 4 describes the intervention characteristics (full details available in supplementary material *S3*). All included studies used different exergaming interventions; Xbox Kinect (Benzing & Schmidt, 2019), Nintendo Wii Sport (Chang et al., 2022), Virtual Reality incorporated cognitive behavioural therapy (VR-CBT) (Chu et al., 2023), and BrainFit (interactive augmented reality-based exercise therapy) (Zhao et al., 2024). Participants in the control groups received Learning Style Profile (LSP) intervention (*n* = 1) (Chu et al., 2023), no intervention (*n* = 1) (Chang et al., 2022), or were placed on a waiting list (*n* = 2) (Benzing & Schmidt, 2019; Zhao et al., 2024).

**Table 4.** Intervention characteristics.

Study	Type of exergame	Exergame features	Duration	Components of fitness	Control group	Measures of EF
Benzing and Schmidt (2019)	Xbox Kinect	Single mode; a motion‐sensing; automatically adjusting difficulties	3 × 30 min; 8 weeks	Strength, coordination, endurance	Waiting list	Simon task; Colour span backward task; Flanker task
Chang et al. (2022)	Nintendo Wii Sport	Single and versus mode; a motion‐sensing	3 × 60 min; 12 weeks	Coordination	None	Stroop test
Chu et al. (2023)	VR-CBT	Single mode; a motion‐sensing	2 × 20 min; 20 weeks	Speed reaction, endurance	LSP	Go/No Go Task
Zhao et al. (2024)	BrainFit	Single mode; a motion-sensing; automatically adjusting difficulties	3 × 30 min; 4 weeks	Coordination, speed reaction	Waiting list	BRIEF

### Risk of bias in studies

**Figure 2.**
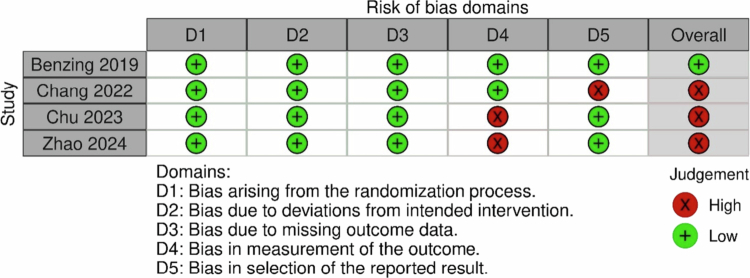
Risk of bias assessment.

The RoB assessment is presented in Figure 2. One study was rated as having low RoB (Benzing & Schmidt, 2019). Most biases related to the measurement of the outcome data because of using a parent-reported form (Zhao et al., 2024) and not specifying whether the test was computerised or not (Chu et al., 2023). Bias due to selection of the reported results was found in one study due to missing reaction time data as was specified in the study protocol (Chang et al., 2022).

### Results of individual studies and syntheses

#### Vote counting

The results of vote counting based on direction of effects in the included studies found that exergaming has positive direction of effects on overall EFs (2 of 2 studies) and inhibition (2 of 3 studies), whereas the studies investigating working memory and cognitive flexibility found inconsistent effects of exergaming (Table 5).

**Table 5.** Vote counting based on direction of effects.

Study	Overall executive functions	Inhibition	Working memory	Cognitive flexibility
Benzing and Schmidt (2019)	Not Relevant	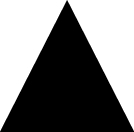	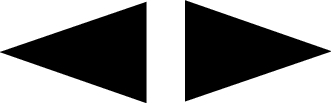	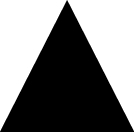
Chang et al. (2022)	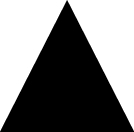	Not Relevant	Not Relevant	Not Relevant
Chu et al. (2023)	Not Relevant	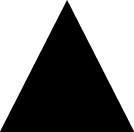	Not Relevant	Not Relevant
Zhao et al. (2024)	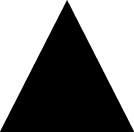	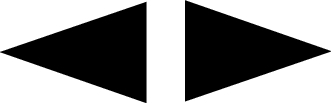	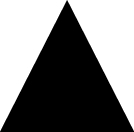	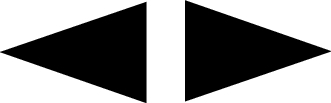
Overall	2 of 2 studies (100%) 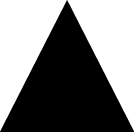	2 of 3 studies (67%) 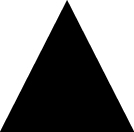	1 of 2 studies (50%) 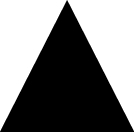	1 of 2 studies (50%) 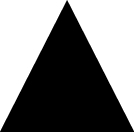

Note: 
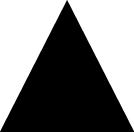
 Positive direction of effect (beneficial); 
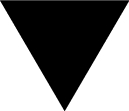
 negative direction of effect (adverse event); no clear effect; 
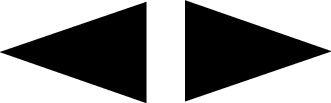
 Not relevant = not included as an outcome of the study.

#### Meta-analysis

Two studies (Benzing & Schmidt, 2019) and (Zhao et al., 2024) were included in the meta-analyses because those studies reported effect sizes on inhibition, working memory, and cognitive flexibility which were measured using valid and reliable outcome measures; inhibition (Simon Task, Behaviour Rating Inventory of Executive Function (BRIEF)), working memory (Colour Span Backward Task, BRIEF), cognitive flexibility (Flanker Task, BRIEF). We could not include studies by Chang et al. (2022) and Chu et al. (2023) in meta-analyses because of incomplete outcome data.

**Figure 3.**
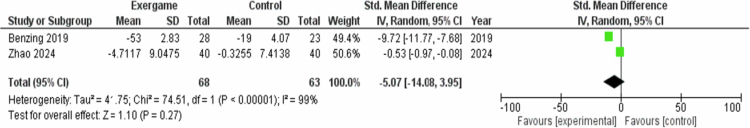
Forest plot of the effects of exergaming intervention on inhibition.

Figure 3 illustrates the forest plot of the meta-analysis for all included studies. Negative SMD indicated a beneficial effect of inhibition, working memory, and cognitive flexibility. The overall effect of exergaming interventions on inhibition processes showed a very large effect size favouring the intervention (SMD = −5.07; 95%-CI [−14.08, 3.95]; *p =* 0.27), which was not significant. The 95%-CI is very wide, compatible with both very large positive effects and negative effects. There was considerable heterogeneity (I² = 99%).

**Figure 4.**
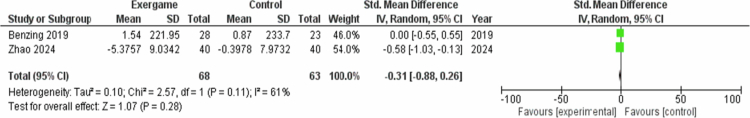
Forest plot of the effects of exergaming intervention on working memory.

As illustrated in Figure 4, the overall effect on working memory was a small to medium effect favouring the intervention (SMD = −0.31; 95%-CI [−0.88, 0.26]; *p =* 0.28). This was not significant. The 95%-CI is very wide compatible with both large positive effects and small negative effects. Heterogeneity was substantial (I² = 61%).

**Figure 5.**
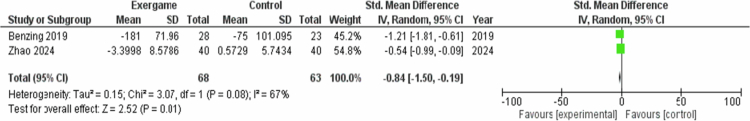
Forest plot of the effects of exergaming intervention on cognitive flexibility.

The overall effect of exergaming interventions on cognitive flexibility as shown in Figure 5 was large and statistically significant (SMD = −0.84; 95%-CI [−1.50, −0.19]; *p =* 0.01). The 95%-CI is compatible with a very wide range of positive effects from very large to small. There was substantial heterogeneity (I² = 67%).

All results from meta-analyses had a very wide CI and crossed the minimal clinically important difference (MCID) for inhibition (Hedges’ g = −0.35), working memory (Hedges’ g = −0.38), cognitive flexibility (Hedges’ g = −0.35) (Isfandnia et al., 2024).

#### Adherence to exergaming intervention

The dropout rate of the participants across the included studies varied ranging from 0% (Chang et al., 2022) to 21.43% (Benzing & Schmidt, 2019), with a mean drop-out rate of 13.82%. Table 6 presents the detailed drop-out rate.

**Table 6.** Drop-out rate in exergaming interventions.

Study	Baseline intervention group (*n*)	Drop-out participants in the intervention group (*n*)	Drop-out rate	Reasons
Benzing and Schmidt (2019)	28	6	21.43%	6 did not want to continue training and participate in the post-test
Chang et al. (2022)	16	0	0%	–
Chu et al. (2023)	39	7	17.95%	2 lost to follow up; 3 Covid-19; 1 insufficient time; 1 unknown reason
Zhao et al. (2024)	40	4	10%	(1 lost interest, 2 withdrew consent, 1 discontinued due to illness)
Total	123	17	13.82%	

#### Implementation strategy

All included studies did not report the implementation strategy based on the StaRI checklist (Pinnock et al., 2017; supplementary material *S4*). All studies reported resources which should be available to facilitate implementation the intervention, including physical infrastructure and staff resources. However, none of the studies reported the process and evaluation of the implementation by the recipients and practitioners in a real-world setting, including economic context, barriers of implementation, cost-effectiveness, and fidelity to implementation strategy (Table 7). Game licences, Xbox Kinect/Nintendo Wii/BrainFit/VR-CBT, television (TV), a spacious room, and a trainer/parent were reported as facilitators of exergaming.

**Table 7.** Implementation strategy.

Implementation strategy							
Study	Context in which the intervention was implemented				Costs use	Fidelity to implementation strategy	Important harms or unintended effects in each group
	Setting	Economic	Barriers	Facilitators			
Benzing and Schmidt (2019)	Home-based	–	–	Xbox Kinect; Shape-up game license; TV; room; parents	–	–	Slight muscle; joint ache; eyestrain
Chang et al. (2022)	School-based	–	–	the Nintendo Wii Sport; game license; a TV set with a 55-inch LCD; panel (Chimei, TL-55LV700D, 1920*1080); room; trainer	–	–	–
Chu et al. (2023)	Laboratory-based	–	–	A desktop computer; a light-emitting diode floor tile screen (3m x 3m) with a resolution of P3.91, surrounded by guardrails and walls; a therapist	–	–	–
Zhao et al. (2024)	Hospital-based	–	–	BrainFit software; iPad technology; Phone tripod; room; a trainer	–	–	–

#### Reporting biases

All registered trials related to the effects of exergaming on EFs in children with ADHD were tracked. At the time of this study, some registered trials were already completed and published while others were still in-progress. Therefore, we assumed no publication bias on this topic.

#### Certainty of evidence

All data from three meta-analyses of the outcomes (inhibition, working memory, and cognitive flexibility) and data from vote counting based on direction of effects on the overall EFs were assessed using GRADE and resulted as being of low quality, except for inhibition which has moderate quality. All detailed results are presented in Table 8.

**Table 8.** GRADE assessment.

	Certainty assessment							Effect	Certainty	
No of studies	Risk of bias	Inconsistency	Indirectness	Imprecision	Publication bias	Large effect	Exergame	Control	(95% CI)	Quality
Inhibition (*N* = 2)	Serious	Serious	Not serious	Serious	Undetected	Very large	68	63	SMD **5.07 lower** (14.08 lower to 3.95 higher)	⨁⨁⨁◯ Moderate
Working memory (*N* = 2)	Serious	Serious	Not serious	Serious	Undetected	–	68	63	SMD **0.31 lower** (0.88 lower to 0.26 higher)	⨁⨁◯◯ Low
Cognitive flexibility (*N* = 2)	Serious	Serious	Not serious	Serious	Undetected	Large	68	63	SMD **0.84 lower** (1.5 lower to 0.19 lower)	⨁⨁◯◯ Low
Overall EFs (*N* = 2)	Serious	Serious	Not serious	Not serious	Undetected	–	56	56	–	⨁⨁◯◯ Low

## Discussion

### Key findings

This is the first systematic review and meta-analysis to synthesise the literature on the effects of exergaming on EFs in children with ADHD. The overall effects of meta-analyses of two included studies demonstrated that there is insufficient evidence to determine whether exergaming improves EFs; although the direction of effects trends positively, the estimates are highly imprecise. While our meta-analyses found heterogeneous and nonsignificant effects of exergame intervention on particular domains of EFs. However, these results should be interpreted with caution given that few studies met inclusion criteria, limiting statistical power (IntHout et al., 2015). These non-significant effects should not be interpreted as evidence of no benefit, particularly given the tendency towards positive effects identified through our vote counting (Altman & Bland, 1995; Gartlehner et al., 2023). Overall, whereas exergaming presents promise direction of effects for enhancing overall EFs and each domain of EFs (inhibition, working memory, and cognitive flexibility), the current evidence is insufficient and highly imprecise to draw definitive conclusions.

### Findings on executive functions

Findings from the vote counting approach suggests that exergaming has positive direction of effects on overall EFs. However, the data are not statistically robust enough to determine a clear effect of exergaming on EFs. Those findings are similar to the primary study by Flynn et al. (2014) investigating the effects of exergaming on EFs in children without ADHD, which found a significant beneficial effect on overall EFs. These imply that although the direction of effects is favourable, there is not enough evidence to declare whether exergaming can improve EFs.

Previous systematic review and meta-analysis on the effects of exergaming on EFs in typical children found that exergaming has moderate beneficial effects for inhibition, and small to moderate beneficial effects for working memory and cognitive flexibility (Chen et al., 2023). Our systematic review and meta-analysis focussed on children with ADHD had the same beneficial direction of effect with a large effect size for inhibition and small to moderate effect size for working memory albeit not statistically significant. While statistically significant large effect for cognitive flexibility. Shuai et al. (2021) provides one plausible explanation in that a low initial level of EFs leads to children with ADHD to obtain higher effects from exergaming interventions than children without ADHD. Moreover, the non-statistically significant results in the current meta-analysis may be attributable, in part, to the small number of studies included (Schünemann et al., 2019).

Furthermore, all findings in each meta-analysis of this study had large CIs, impacted by a limited number of studies included in meta-analysis (Schünemann et al., 2019). All findings in meta-analyses cross the MCID of inhibition, working memory, and cognitive flexibility of the previous systematic review on the effects of medication (methylphenidate) on EFs in children with ADHD by Isfandnia et al. (2024). Those reveal that although the exergaming can potentially be an effective alternative non-pharmacological treatment for EFs in children with ADHD; it remains uncertain due to the estimate is notably imprecise.

Since sub-group, sensitivity analysis, and meta-regression could not be performed due to the limited number of studies included in each meta-analysis, we performed a qualitative investigation of methodological and clinical factors. We assume that the differences of intervention characteristics, such as type of exergaming (Manser et al., 2024); duration (Singh et al., 2025); components of fitness (Chen et al., 2024; Singh et al., 2025); and measures of EFs (Singh et al., 2025), and the bias arising from the use of parent reported cognitive measurement in the study conducted by Zhao et al. (2024) could be the cause of the heterogeneity. As found in the previous study, cognitive measurement from patient reported could result in bias in terms of collection; non-response; proxy; recall; language; fatigue; and timing (Zini & Banfi, 2021).

### Findings on the adherence to exergaming

Current findings suggest that the adherence to exergaming interventions in children with ADHD is high, with approximately 14% average drop-out rates. These results are in line with the previous findings by Caselles-Pina et al. (2023), which suggested that adherence to video game-based interventions is high with drop-out rates ranging from 5% to 22.35%. Furthermore, a previous systematic review by Vancampfort et al. (2016) found that there were 17.5% (9.8% to 29.4%) drop-out rates in general PA treatments. These data suggest that exergaming interventions might have slightly lower drop-out rates compared to general PA interventions and can potentially be an effective approach to increase adherence to PA in this population.

### Findings on implementation strategy of exergaming

Available studies provided evidence on the potential implementation of exergame interventions across settings, including home, school, and health-care facilities, which aligned with the guidelines on managing children with ADHD (Wolraich and Hagan, 2019). Several potential harms were also identified by available studies. However, the influence of the potential harms on the acceptability of the exergame intervention by both recipients, parents, and practitioners were not informed by available studies. In addition to information on the resources that need to be available to facilitate implementation, the uptake of the evidence into routine practice requires information on both the process and evaluation of the implementation by practitioners (Koorts et al., 2018). Further primary studies informed by process models, evaluated using evaluation frameworks, and reported in adherence with implementation research reporting guidelines are needed to provide evidence and to facilitate the uptake of evidence by practitioners in routine practice (Nilsen, 2015).

### Strengths and limitations

This systematic review used comprehensive search strategies and electronic databases which were consulted with an academic librarian to verify the specificity and sensitivity (Asubiaro & Isioma, 2022; Rethlefsen et al., 2015). Moreover, this systematic review implemented rigorous and robust methods by following the Cochrane Guidelines for Systematic Reviews, PRISMA Guideline, SWiM Guideline, StaRI Guideline, a published study protocol, and involving two researchers in independent screening, data extraction and study quality assessment processes.

Nevertheless, there are some limitations. Not all study authors responded to missing data requests meaning that the meta-analyses contained only two out of four eligible studies included in the review. Therefore, we could not assess the heterogeneity through sub-group analysis, or not perform sensitivity analysis, or not conduct meta-regression, or assess publication bias, and only derived previous studies to deduce the reason. Secondly, we were unable to formally assess publication bias. Prior evidence syntheses suggest that exergame interventions for individuals with neurocognitive impairment are unlikely to be strongly affected by publication bias (Chan et al., 2024; Maggio et al., 2025), although indications of bias have been reported in the broader exergame literature (Chen et al., 2023). To mitigate this risk, we have already searched clinical trial registries. Given the presence of ongoing trials, future updates of systematic review should track these studies and incorporate their results even if only registry outcomes are available prior to full publication. Thirdly, the measures of EFs vary across studies which pooled objective computerised EFs tests with parent-rated on the BRIEF. Therefore, it complicates pooled analysis resulting in high heterogeneity and limit interpretability and comparability. These severely limit the statistical power of our findings and restrict the generalisability of any conclusions across the spectrum of children with ADHD. Therefore, our findings strengthened previous evidence syntheses calling for the development and validation of assessment tools for EFs that can be generalised across contexts (Dias et al., 2024; Kusi-Mensah et al., 2022; Souissi et al., 2022; Nyongesa et al., 2019). Moreover, future study should use consistent EFs measurement modalities. Fourthly, most eligible studies were at high risk of bias, which may impact the significance of the analysis. Because of limited eligible studies for the review, studies with high risk of bias were included in the meta-analysis, which needs to be considered during interpretation of the results. Well-designed and well-reported RCTs are needed to investigate the effects of exergaming on EFs in children with ADHD. Future research should comply with the relevant reporting guidelines, for example, CONSORT-EHEALTH, to clearly report the measurement of the outcome, and should completely report the outcomes based on the registered protocol. Lastly, the included studies did not report most aspects of their implementation strategies and results, for example, cost-effectiveness. Therefore, our review cannot provide the real-world feasibility across different settings and scalability for the future implementation, especially in terms of cost-effective expansion. The consideration of how exergaming could be implemented in societies is crucial. Future primary studies should therefore consider implementation strategy in detail, including the exergame characteristics by adopting both specific exergame framework, such as Beyond ‘just’ fun framework (Manser et al., 2025) or Multidisciplinary Iterative Design of Exergame Framework (Li et al., 2020), and implementation science frameworks and guidelines, such as, Consolidated Framework for Implementation Research (CIFR) (Damschroder et al., 2022) and StaRI (Pinnock et al., 2017), to ensure that the study is well reported and use a valid conceptual implementation strategy frameworks (Curran et al., 2012).

### Recommendations and implications

While exergaming interventions present positive direction of effect to increase overall EFs, inhibition, working memory, cognitive flexibility, and adherence to PA, the current study is too limited and highly imprecise to determine whether exergaming improves EFs. Therefore, parents at home and professionals working in laboratories, schools, or hospitals could adopt exergaming as an adjunct or complementary treatment for EFs and adherence to PA. Further primary studies following implementation science guidelines are needed to both provide higher quality evidence on the effectiveness and facilitate the uptake of evidence by practitioners in routine practice across settings to improve management of children with ADHD.

## Conclusion

This study suggested that current evidence cannot provide a definite conclusion since there is insufficient evidence to determine the effects of exergaming on EFs. Whilst there is promising evidence that exergaming may be an effective adjunct or independent treatment for ADHD in children, three of four studies had a moderate-to-high risk of bias and combined markedly heterogeneous interventions (exergaming formats and durations) and non-comparable EF outcomes (computerised test vs parent-reported scales). Together with the very high I² values, this suggests that the pooled estimates may not represent a single underlying effect and may instead be driven by measurement differences and imprecision. We planned subgroup and sensitivity analyses to explore this heterogeneity, but the small number of available studies precluded these analyses. Therefore, quantitative synthesis must be interpreted with great caution, and the resulting estimates may have limited clinical or theoretical meaning. Moreover, exergaming has the potential to be implemented across different contexts and settings. Future research with high-quality measurement of the outcome and completely reporting outcome data is necessary to strengthen the evidence, to allow analysis of heterogeneous results, and to improve the reliability and generalisability of the findings. Additionally, research in the implementation strategy of exergaming is crucial to allow deeper analysis on how exergaming could be implemented.

## Supplementary Material

Supplementary Material S2 SWiM Checklist.docx Supplementary Material S2 SWiM Checklist.docx

Supplementary Material S3 Study Characteristic.xlsx Supplementary Material S3 Study Characteristic.xlsx

Supplementary Material S1_PRISMA 2020 checklist.docx Supplementary Material S1_PRISMA 2020 checklist.docx

Supplementary Material S4 StaRI checklist.xlsx Supplementary Material S4 StaRI checklist.xlsx

## Data Availability

The data presented in this study are available upon reasonable request.

## Open materials badge

Preregistered: https://www.crd.york.ac.uk/PROSPERO/view/CRD42024549395.
